# Fluoride content and recharge ability of five glassionomer dental materials

**DOI:** 10.1186/1472-6831-8-21

**Published:** 2008-07-28

**Authors:** Dejan Lj Markovic, Bojan B Petrovic, Tamara O Peric

**Affiliations:** 1 Faculty of Dentistry, Clinic for Paediatric and Preventive Dentistry, Dr Subotica 11, Belgrade, Republic of Serbia; 2 Dentistry Clinic of Vojvodina, Department for Paediatric and Preventive Dentistry, Hajduk Veljkova 12, Novi Sad, Republic of Serbia

## Abstract

**Background:**

The relationship between fluoride content and fluoride release for glass-ionomer cements is not well understood. The aim of this laboratory study was: to determine the fluoride concentrations at the surfaces of glass-ionomer materials with respect to different storage media and different pH environments; to examine the recharge ability of the materials after NaF immersion; and to assess the morphological changes at the material surfaces using scanning electron microscope and energy dispersive spectroscopic techniques (SEM/EDS).

**Methods:**

Five glass-ionomer materials, Fuji Triage (FT), Fuji II LC (FII), Fuji VIII (FVIII), Fuji IX GP (FIX), and Ketac N100 (KN), were analyzed in this study. Resin-based fluoride releasing material Helioseal F (HSF) was used as a comparison material. The sample consisted of 120 cured cement disks (n = 20 disks of each tested material, 10 × 1.5 mm). Five disks of each material were stored in 4 different storage media (I- saline, II- acidic solution ph = 2.5, III- acid solution ph = 5.5, IV- NaF solution (c = 500/106). After 7 days, two disks of each material were transferred from media I, II and III to the NaF solution for 3 min. EDS analysis was conducted in 3 randomly selected spots of each experimental disk. SEM was used to determine morphological characteristics of the material surface. Differences between the experimental groups have been analyzed using Student's t-test with the level of significance set at p < 0.001.

**Results:**

FT showed the highest fluoride content at the surface of the material. The lowest amounts of fluoride ions were detected at the surfaces of the FT disks stored at low pH environments, and this difference was statistically significant (p < 0.001). Glass-ionomers showed significantly higher fluoride concentrations when compared to the HSF (p < 0.001). After immersion in the NaF solution, fluoride concentrations at the surfaces of the disks increased when compared with previous storage media (FT>FVIII>KN>FII>FIX). SEM analysis of the surface morphology revealed numerous voids, cracks and microporosities in all experimental groups, except for KN and HSF. More homogenous material structure with more discrete cracks was observed in samples stored at neutral pH environment, compared to disks stored in acidic solutions.

**Conclusion:**

The tested materials could be considered as promising dental materials with potential prophylactic characteristics due to their relatively high fluoride content, but also the ability to extensively reabsorb fluoride ions, especially in acidic environments.

## Background

Modern approach to the control of dental caries requires dental materials which possess both restorative and prophylactic characteristics. The anticariogenic behaviour of a dental material has been attributed to its fluoride content [1]. The fluoride content in the material, as well as the amount of released fluoride necessary for "curing" carious lesion and for prevention of secondary caries, have not been well documented. It may be assumed that the content of fluoride should be as high as possible, yet without adverse effects on the physical properties of the material. It has been shown that if a dental material exhibited high fluoride release, it had inferior mechanical properties [2].

Glass-ionomer cements are characterized by acid-base setting reaction, chemical bonding to enamel and dentine, fluoride release, biocompatibility and acceptable aesthetics [3,4]. Generally, it can be assumed that a major advantage of glass-ionomers is their potential cariostatic effect [5], due to the fluoride release [4] and antibacterial activity [5,6]. Glass-ionomer cements contain 10 to 23% fluoride [7]. In general it may be supposed that there is a direct relationship between the fluoride present in the cement and the amount of fluoride released [8-10].

Laboratory studies [1,11] clearly demonstrated strong effects of glass-ionomers on caries development and progression. The data collected in these studies suggest that fluoride release from dental materials is dependent on the medium used in the evaluation. Storage at low pH environments accelerates the amount of fluoride released from glass-ionomers, suggesting a strong anticariogenic potential in real clinical situations. However, clinical investigations showed contradictory results with regard to caries development. Many clinical trials reported significantly lower incidence of secondary caries around glass-ionomers compared with other restorative materials [1,12]. Nevertheless, other studies revealed relatively high frequency of secondary caries in relation to failures of glass-ionomer restorations in general dental practice [13-15].

Today, there is a variety of glass-ionomer materials available in the market. The purpose of this study was:

- to determine the fluoride concentrations at the surfaces of glass-ionomer materials with respect to different storage media and different pH environments;

- to examine the recharge ability of the materials after NaF immersion; and

- to assess the morphological changes at the material surfaces using SEM/EDS.

## Methods

Five glass-ionomer materials, Fuji Triage Capsule (FT, conventional glass-ionomer protection material, GC Int, Tokyo, Japan), Fuji IX GP (FIX, conventional glass-ionomer restorative cement, GC Int), Fuji VIII GP Capsule (FVIII, resin-modified glass-ionomer restorative cement, GC Int), Fuji II LC Capsule (FII, light-cured resin modified glass-ionomer restorative material, GC Int) and Ketac N100 (KN, light-cured nano-ionomer restorative material, 3 M ESPE AG, Seefeld, Germany), were analyzed in this study. Helioseal F (HSF, resin-based fluoride releasing material, Ivoclar Vivadent AG, Schaan, Liechtenstein), was used as a comparison material.

Tested materials were prepared according to the manufacturers' instructions. The capsulated glass-ionomer materials (FT, FII and FVIII) were activated just before mixing, set into the amalgamator, and mixed for 10 seconds at high speed. The mixed capsules were loaded into the capsule applier. The powder and liquid components of the FIX were dispensed onto the pad using the plastic spatula. The powder was divided into 2 equal parts. The first portion was mixed with all the liquid for 10 seconds. The remaining portion was incorporated and mixed thoroughly for 15–20 seconds. Two KN pastes were dispensed onto the mixing pad and mixed together for 20 seconds using the plastic cement spatula until the uniform colour was achieved. HSF was used directly and dispersed with the disposable cannula.

Immediately after mixing the materials were transfered into a cylindrical teflon mould (10 × 1.5 mm). During setting, the bottom and top of the moulds were covered by glass plates using hand pressure. Light cured materials (FII, KN, HSF) were photoactivated for 40 seconds with photopolimerisation device (Blue Lex LD-105, Monitex Industrial Co, Taipei, Taiwan). The setting reaction of FT was accellerated with a 40s photo-activation. FVIII and FIX samples were maintained inside the moulds, covered by the matrix, for ten minutes.

After hardening, the specimens were removed from the mould and all discs were submitted to standard polishing under wet conditions using Sof-Lex discs 8691-F (3 M ESPE AG, Seefeld, Germany). After polishing, the disks were transferred to 10 ml of deionized water and stored at 37°C. The whole sample, consisted of 120 disks (n = 20 disks of each material) was submitted to 4 different storage media (n = 5 disks of each material per each storage medium). The storage media were prepared as follows:

- medium I – 10 ml of saline

- medium II – 10 ml of acidic solution at pH 2.5 made of a 0.1 M lactic acid solution acidified with HCl;

- medium III – 10 ml of acidic solution at pH 5.5 made of 50 mmol/l KCl titrated to pH 5.5 with concentrated HCl

- medium IV – NaF solution (c = 500 ppm)

Disks were stored in four different storage media at 37°C for 7 days. After that period, 2 disks of each material were transferred from media I, II and III to the NaF solution (containing 500 ppm of F). Those samples were immersed for 3 min to simulate the fluoride ion recharge.

The surface of each disk was rinsed with 2 ml of deionized water. The specimens were mounted on aluminium stubs, sputter-coated with gold (Bal-Tec SCD 005 Sputter Coater, Bal-Tec AG, Balzers, Liechtenstein), and then examined using scanning electron microscope (Jeol JSM-6460LV, Jeol Industries Ltd., Tokyo, Japan) equipped with energy dispersive spectrometer (SEM/EDS). In each disk, SEM/EDS analysis was performed in three randomly selected spots. Quantitative changes of the material surface and the recharge ability after the NaF treatment were evaluated using EDS. SEM was used to determine the effects of different storage media and different pH environments on morphological characteristics of cured cement disks. The criteria for evaluation included the presence of cracks and micropores at the surface of the material.

Descriptive statistical analyses were primarly performed. Differences between the experimental groups has been analyzed using Student's t-test, with the level of significance set at p < 0.001.

## Results

### Surface concentration of fluoride in relation to the storage medium

A total of 84 disks were analyzed (3 disks of each material from media I, II and III, and 5 disks of each material from medium IV). Table 1 shows average values of the fluoride surface concentrations (in mass% of F- wt%) in relation to storage medium. In all storage media, surfaces of glass-ionomer materials showed significantly higher fluoride concentrations when compared to the HSF (p < 0.001 Student's t-test). The lowest concentrations of fluoride ions were detected at the surfaces of disks stored in acidic environment at pH = 2.5. Significant differences (p < 0.001 Student's t-test) in fluoride concentrations were observed in relation to storage media (IV>I>II>III).

**Table 1:** Surface concentration of fluoride in relation to the storage medium.

	medium I		medium II		medium III		medium IV	
	wtF(%)	SD	wt F(%)	SD	wt F(%)	SD	wt F(%)	SD

FT	10.0^a,b,c,d^	0.56	3.8^a,b,c,d^	0.25	4.8^a,b.c.d^	0.20	15.6^a,b,c,d^	0.40
FII	7^A,B,C,D^	0.25	4.6^A,B,D^	0.22	4.6^A,C,D^	0.16	7.8^A,B,C,D^	0.26
FVIII	10.4^x,y.w.z^	0.30	4.8^x,y,w,z^	0.26	6.4^x,y,w,z^	0.39	11.5^x,y,w,z^	0.52
FIX	10.1^X,Y,W,Z^	0.49	8.2^X,Y,W,Z^	0.56	9.6^X,Y,W^	0.39	9.5^X,Y,Z^	0.42
KN	7.6^1,2,3,4^	0.39	2.6^1,2,4^	0.46	2.5^1,3,4^	0.35	8^1,2,3,4^	0.65
HSF	1.4^I,III,IV^	0.39	1.2^II,IV^	0.27	1^I,III,IV^	0.26	2.5^I,II,III,IV^	0.48

The F concentrations (wt%); n = 3 disks for media I, II, III, n = 5 disks for medium IV; SD; same superscripts indicate average values with statistically significant differences (p < 0.001);

### The effect of fluoride immersion

The effect of NaF immersion on fluoride content at the surface of the glass-ionomer material was evaluated in 36 disks (2 disks of each material from media I, II, and III). For all tested materials, fluoride concentrations at the surfaces of the disks increased when compared with the previous storage media (table 2).

**Table 2:** The effect of NaF immersion.

	Disks previously stored in medium I		Disks previously stored in medium II		Disks previously stored in medium III	
	wtF(%)	SD	wt F(%)	SD	wt F(%)	SD

FT	15.7	0.37	15.5	0.31	15.5	0.20
FII	7.8^A,B.C^	0.25	8.5^A,B^	0.36	8.25^A,C^	0.27
FVIII	12.1^x,z^	0.37	12.5^y,z^	0.37	10.5^x,y,z^	0.37
FIX	11.4^X,Z^	0.37	11.8^Y,Z^	0.37	10.5^X,Y,Z^	0.37
KN	8.2	0.69	8.2	0.52	8	0.70
HSF	2.5	0.44	2.67	0.52	2.75	0.72

The F concentrations (wt%); n = 2 disks; SD; same superscripts indicate average values with statistically significant differences (p < 0.001).

### SEM analysis of the surface morphology

**Figure 1:**
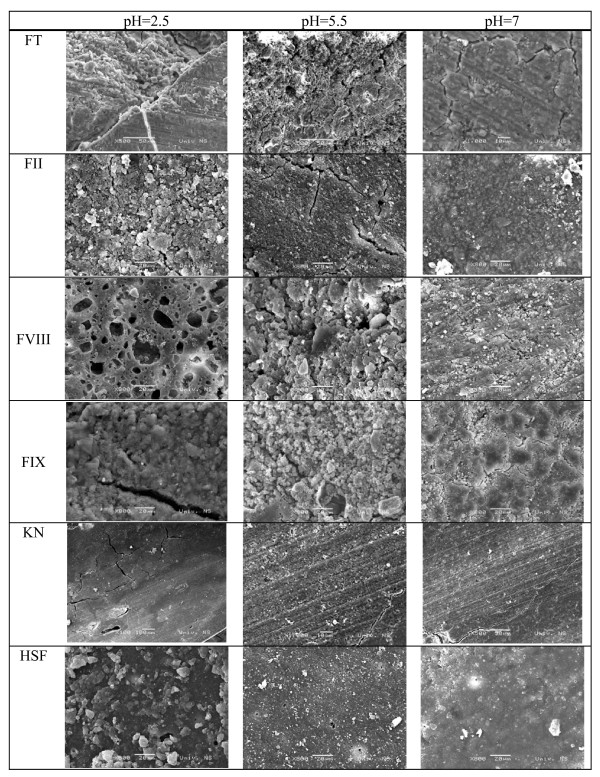
Surface morphology in relation to storage media (SEM images of tested materials at 500×–1000× magnification).

SEM showed morphological differences in all materials, in the samples stored at pH 2.5, pH 5.5 and in saline (figure 1). More homogenous material structure with more discrete cracks was observed in samples stored at neutral pH environment. Destruction of the material surface was evident in samples stored at pH 2.5. SEM analysis revealed the presence of a large number of voids, cracks and microporosities in FT, FII, FVIII and FIX specimens at pH 2.5, yet they were not detected in KN and HSF specimens.

## Discussion

After more than 30 years of use, glass-ionomer materials continue to be popular for different indications in contemporary dentistry. Fluoride is an essential component of contemporary dental materials, including glass-ionomer cements. Fluoride is used as a flux during glass manufacture with the specific purpose of leaching fluoride into the surrounding tissues in order to provide caries prevention or secondary caries inhibition. Glass ionomers release significant amounts of fluoride into tooth structure, as well as into the oral environment. During the caries process, an acidic environment attacks dental tissues as well as the glass-ionomer cement [16]. In the present study two conventional glass-ionomers (FII and FIX), two resin modified glass-ionomers (FII and FVIII), and a nano-ionomer (KN) were evaluated. Fluoride releasing resin based fissure sealant (HSF) was used as comparison material. The materials were chosen as being widely used in contemporary dental practice.

The extended use of experimental models for glass-ionomers evaluation and the extrapolation of the *in vivo *performance of materials based on the results obtained from laboratory tests have raised numerous concerns on the clinical relevance of laboratory testing protocols [17]. The present study is preliminary investigation limited on several distinct parameters. Only controlled clinical trials, as well as more complex experimental studies comprising large number of factors that may influence the properties of dental materials in real clinical situations could provide more valid conclusions.

The majority of the research into release and uptake of fluoride ions have utilised analysis of ion concentrations in solutions after glass-ionomer immersion [4,19-21]. Useful information have been obtained about the influence of the initial glass-ionomer composition on the removal of ions from solution and their subsequent re-release pattern. However, this approach provides modest information about the morphological and compositional changes that occur in the material during immersion.

Great differences in fluoride release between various materials have been described [22]. However, the relationship between fluoride content and fluoride release for glass-ionomer cement is not well understood. Kuhn and Wilson [23] indicated the existence of three mechanisms concerning fluoride release from glass-ionomers: superficial rinse, diffusion through pores and micro-fractures and finally mass diffusion. The fluoride content at the surface of the glass-ionomer material is an important parameter in quantifying the recharge ability of the glass-ionomer. Hadley has demonstrated, using depth profiling, that the concentration of fluoride maximised at the surface of the samples [24].

The importance of the knowledge of fluoride release in different storage media has increased in the last years. In a real clinical situation, during the caries attack, acidogenic bacteria create an acid micro-environment that may modify cement's properties [25,26]. The model used in the present study focused on assessment of quantitative and qualitative changes at the surfaces of different glass-ionomer materials.

In the first part of the experiment the fluoride concentration at the surface of the materials with regards to different storage media was evaluated. We hypothesized that different storage media and different pH environments significantly affect the fluoride concentration at the material surface and the rate of fluoride release from a glass-ionomer cement. The quantity of released fluoride was found to be proportional to the fluoride content of the glass-ionomer and other fluoride containing dental materials [27,28]. That is why the fluoride concentration was used as a parameter for anticipation of fluoride release in the present study. The fluoride content observed in this study appeared to be significantly lower, compared to some previous investigations and the claims of the manufacturers [7]. Value of the highest fluoride content measured was 19%. The reasons for this discrepancy may be found in the experiment design, but also in the relatively low sensitivity of the EDS analysis, which is a semiquantitative analytical method. Results obtained in this study clearly show that fluoride concentration at the material surface is strongly dependent on storage medium for all tested materials. Important differences between tested materials concerning fluoride concentration were recorded. FT and KN exhibitted the lowest amounts of fluoride in acidic environment compared to the baseline fluoride concentrations observed in saline. In fact, FT released greater amount of fluoride compared to other tested materials. FT, FVIII and FIX exhibited lower amount of fluoride at pH = 2.5 than at pH = 5.5 or pH = 7. Results of the present experimental study are in agreement with finding that glass-ionomer releases more fluoride when the environment was at lower pH, leaving less fluoride content at the surface of the material stored at the acidic solution, thus providing the highest amount of fluoride when it is most needed to prevent secondary caries [7].

The ability to take up and re-release ions from solution is an important asset of glass ionomer cements, which may allow their application as 'rechargeable reservoirs' for the distribution of ions, including fluoride [21]. Despite the substantial number of researches aimed at establishing the mode of fluoride uptake and re-release [29], the underlying mechanism remains unclear. Most of the researches into fluoride ion uptake have utilized analysis of ion concentrations in solution before and after GIC immersion [1,8,19,21,22,29]. In the second part of the present study, the ability of fluoride treatment was tested through immersion of samples into NaF solution containing 500 ppm. The main objective was to determine the fluoride absorption ability of the material stored under the different conditions. In agreement with previous studies, the present results showed that exposure of glass-ionomer materials, as well as resin- based fluoride releasing fissure sealant to a solution containing fluoride allows the materials to take up fluoride. Fluoride levels increased to five times more (FT) after treatment of glass- ionomer specimens with NaF solutions. The major increase in fluoride content was observed in the disks previously stored in acidic solution. This finding is in line with the previous study which showed that the absorption ability decreases with increase of pH values [22]. Evaluation of ion content at the surface of the material provides usefull information about the influence of the initial material composition on the ion sorption from the solution.

There are some shortcomings of the present experimental model that must be taken into account when comparing results with the similar studies, particularly when extrapolating these results in real clinical situations. Fluoride release was quantified through changes of fluoride concentrations at the surfaces of the materials tested, while the actual fluoride release from glass-ionomers into different storage media has not been obtained. Further on, surface of the material was the central point in the present investigation, without sufficient evidence that the changes at the material surface can represent the changes in the entire restoration.

The SE photomicrographs obtained in this study showed that, regardless of the chemical composition, both conventional (FT and FIX) and resin-modified glassi-onomers (FII and FVIII) presented voids, cracks and microporosities at the disk surface. SEM examination revealed a similar morphological surface pattern to each other. It has been shown that scanning electron microscopy is not a reliable method for assessment of glass-ionomer cements, since glass-ionomers are sensitive to SEM preparation techniques, and the cracks could be produced during specimen processing for SEM analysis [31]. It is possible that there may be some artifacts in the present microstructures of the glass-ionomers observed with the SEM, and future microstructural studies should employ a replica technique in order to capture the changes at the material surface in relation to storage media more precisely. On the other hand, SEM analysis provided direct visibility of the changes at the material surface in relation to storage media. SEM analysis appears to be an efficient and acceptable method of examining such features as surface topography, filler size and distribution, interface adhesion and porosity [32-36]. SEM analysis showed evidence of differences in surface morphology. The presence of more fractures, voids and microporosites were observed in conventional (FT and FIX) and resin-modified glass-ionomers (FII and FVIII) after storage in acidic environment at pH = 2.5. SEM analysis of the specimens stored at pH = 5.5 failed to show clear evidence of differences in surface morphology between the tested materials, although the presence of more fractures at the disks stored in solution with pH 5.5 compared to specimens stored in saline was observed in both conventional and resin-modified glass-ionomers. In conclusion, taking into account the limitations of the methodology employed, from SEM images it is observed that an acid environment is related to the degradation of glass-ionomers according to the results of the recent studies [37]. It is also clear that fluoride release is related to GIC degradation. On the contrary, nano-ionomer material (KN), and resin-based fissure sealant (HSF) appear to be resistant to storage in different pH environment, since no morphological changes at the surface of these materials in relation to storage media could not be observed.

Results of the present study confirm that glassi-onomers are not very resistant to external agents and in low pH environments they are undergoing noticeable destruction. On the other hand, destruction of the material surface is followed by extensive fluoride release necessary to resist the caries attack.

It goes without saying that conclusions from laboratory studies may not be comparable with studies *in-vivo*. In addition, it is important to take into consideration that different methodologies used in the studies, including specimen size, media used to measure fluoride release and uptake, quantity of media used to measure fluoride as well as different methods to measure fluoride release, are responsible for the numerous differences. Thus, comparisons between the materials should be made considering behaviour of materials rather than the absolute amount of released and uptaken fluoride (in absolute terms).

## Conclusion

1. The fluoride concentrations at the surfaces of glass-ionomer based materials is under influence of storage media and pH environments.

2. All tested materials exhibit the property of recharging the fluoride surface concentrations at the material surfaces after fluoride immersion. The increased recharge rate is related to decreasing pH.

3. Acidic environment affects the material surface, resulting in less homogenous structure, with voids, gaps and microporosities. The amount of cracks and microporosities correlates with decreasing pH.

4. All tested materials possess relatively high fluoride content and the ability to extensively reabsorb fluoride ions, especially in low pH environments.

## Competing interests

Dejan Markovic, Bojan Petrovic and Tamara Peric declare that they have no competing interests. Prof. Dejan Markoviæ is the Key Opinion Leader for GC in Serbia. In past five years he received some funding from GC for various scientific activities. GC is not financing this investigation (including the article-processing charge). The authors do not hold any stocks or shares in an organization that may in any way gain or lose financially from the publication of this manuscript. The authors as well do not hold or currently applying for any patents relating to the content of the manuscript, and never had received fundings from an organization that holds or has applied for patents relating to the content of the manuscript. There are no nonfinancial competing interests (political, personal, religious, academic, ideological, intellectual, commercial or any other).

## Authors' contributions

All authors contributed equally to this work. DM, TP and BP have come together to the final conception and design of the study. BP carried out the study at the Department for Scanning Electrone Microscopy, Faculty of Sciences, Novi Sad, and analysed the results. DM, TP and BP have evaluated the morphological changes in material surface together. DM supervised the manuscript, and all authors have given final approval of the version to be published.

## Pre-publication history

The pre-publication history for this paper can be accessed here:


